# Knowledge, attitudes and current practices regarding LI-RADS^®^: A survey from 14 countries in sub-Saharan Africa

**DOI:** 10.4102/sajr.v30i1.3367

**Published:** 2026-02-23

**Authors:** Rajshree Segobin, Dale Creamer, Rufaida Khan, Eduard Jonas, Sanju Sobnach, Sulaiman Moosa

**Affiliations:** 1 Department of Radiation Medicine, Faculty of Health Sciences, University of Cape Town, Cape Town, South Africa; 2 Department of Surgery, Faculty of Health Sciences, University of Cape Town, Cape Town, South Africa

**Keywords:** LI-RADS^®^, knowledge, attitudes, education, sub-Saharan Africa, hepatocellular carcinoma

## Abstract

**Background:**

Hepatocellular carcinoma (HCC) is highly prevalent in sub-Saharan Africa (SSA). LI-RADS^®^ is a standardised system for imaging-based diagnosis and characterisation of HCC.

**Objectives:**

This study assessed knowledge, attitudes and current practices related to LI-RADS in SSA, with a view to identifying barriers to its utilisation and informing targeted educational interventions.

**Method:**

A 21-item anonymous electronic questionnaire was distributed to medical professionals in SSA using the SurveyMonkey online platform. Knowledge, attitudes and current practices regarding LI-RADS^®^ were assessed. Data were analysed using descriptive statistics, and comparisons were made between radiologists and non-radiologists.

**Results:**

There were 134 respondents from 14 of the 34 SSA countries. Radiologists significantly outperformed non-radiologists in LI-RADS^®^ knowledge, particularly regarding its purpose (65.6% vs 38.2%, *p* = 0.0007), arterial phase hyperenhancement definition (90.3% vs 59.8%, *p* = 0.004) and size criteria (77.4% vs 45.1%, *p* = 0.003) for diagnosing hepatocellular carcinoma (HCC). However, 43.8% of radiologists and 63.4% of non-radiologists did not recognise the limitations of LI-RADS^®^. Only 34.3% stated that LI-RADS^®^ was their reporting standard and 29.1% of the respondents indicated that less than 25% of their radiological reports adhered to LI-RADS^®^. The majority (78.3%) of participants stated they preferred radiology reports for high-risk liver lesions to be LI-RADS^®^-standardised. The two main barriers to adopting LI-RADS^®^ included lack of consistency (44.8%) and unfamiliarity with the reporting system (27.6%).

**Conclusion:**

Although LI-RADS^®^ remains the preferred reporting system for HCC, there are significant gaps in its knowledge and implementation across SSA.

**Contribution:**

This survey highlights the needs for targeted educational initiatives and improved training to enhance the adoption and use of LI-RADS^®^ in SSA.

## Introduction

The incidence of hepatocellular carcinoma (HCC) continues to rise globally. By the year 2040, the annual mortality of HCC is expected to reach 1.3 million.^1,2^ In sub-Saharan Africa (SSA), HCC is a public health emergency, and chronic hepatitis B virus (HBV) infection is the main aetiology.^3,4^ The median age at presentation ranges between 28–54 years old, and most patients present with advanced disease. Less than 1% are offered curative-intended therapies, and only 2% are alive at 1 year.^4,5^

Currently, the diagnosis of HCC is almost exclusively made using contrast-enhanced (CE) imaging. In patients with non-cirrhotic tumours and normal serum alpha-fetoprotein (AFP) levels and/or atypical imaging findings, biopsy and histology will confirm the diagnosis.^6,7,8,9,10,11,12^

LI-RADS^®^, developed by the American College of Radiology in 2011, is a quality assurance tool that standardises the reporting of liver lesions at risk of HCC.^13^ Subsequent revisions of LI-RADS^®^ have resulted in a taxonomic system that promotes effective discussion and decision-making in multi-disciplinary teams (MDTs) (hepatologists, surgeons, radiologists, oncologists and palliative care physicians) caring for HCC patients.^9,10,14,15^

Perceived complexity, lack of training and available radiological infrastructure are potential barriers to the use of LI-RADS^®^ globally.^16,17,18,19^ In SSA, it remains unclear whether HCC MDTs have formally adopted LI-RADS^®^. Currently, surveys examining the factors that impact on LI-RADS^®^ utilisation among medical professionals emanate mostly from high-income countries (HICs) and have not been conducted in SSA.^15,16,19,20^

The aim of this study was to describe and analyse the knowledge, attitudes and perceptions, as well as the current practices of medical professionals regarding LI-RADS^®^ in SSA, with a view to identifying barriers to its use and designing and implementing educational programmes on the subcontinent.

## Research methods and design

A previously validated self-administered 21-item anonymous questionnaire was distributed to the target population using the online platform SurveyMonkey (https://www.surveymonkey.com). The survey comprised four sections: (1) seven knowledge-based questions regarding the LI-RADS^®^ lexicon; (2) four questions dealing with the attitudes and perceptions of the respondents towards LI-RADS^®^; (3) three questions addressing the current practises of LI-RADS^®^ by participating respondents at their respective home institutions; and (4) seven demographic questions, including country of practice, hospital setting (academic vs private), specialty, staff grade, experience (years), number of HCC patients seen monthly and number of CT and/or MRI scans reviewed per month. For the purposes of this study, the operational definition of an attitude was a permanent disposition or reaction that represents an individual’s degree of approval or disapproval regarding a particular issue.

A survey web link was thus distributed electronically to registered participants of the Gastroenterology-Extension for Community Healthcare Outcomes (G-ECHO) educational platform of the Gastroenterology and Hepatology Foundation of SSA ahead of a LI-RADS^®^ webinar. Upon completion of the survey, data were extracted using SurveyMonkey tools (https://www.surveymonkey.com) and then imported and stored on a password-locked spreadsheet registry (Microsoft Excel, Redmond, WA, USA). Descriptive statistical methods were used to determine the response rates of the study participants across the various questionnaire categories. Responses were also filtered by specific sub-group (radiologist vs non-radiologist) for sub-group analysis. A questionnaire was deemed complete and suitable for analysis if all sections were completed. Incomplete questionnaires were excluded from the study.

### Ethical considerations

The Human Research Ethics Committee of the Faculty of Health Sciences at the University of Cape Town granted ethical approval for this study (reference numbers: IRB00001938, HREC REF: 961/2023). Institutional permission was further obtained prior to surveying university staff for research purposes.

## Results

**FIGURE 1:**
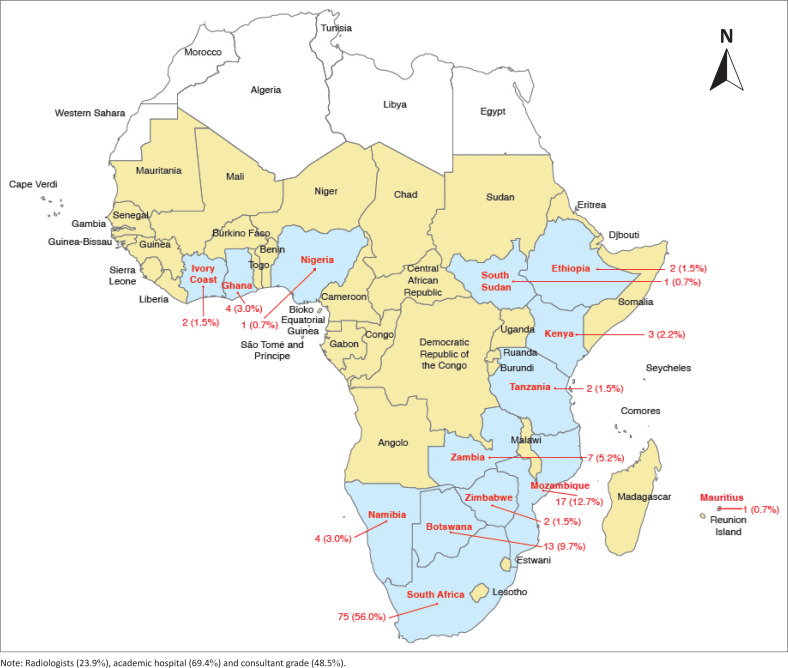
Country of practice of respondents in this study (*N* = 134).

One hundred thirty-six doctors were surveyed. Two respondents submitted incomplete questionnaires and were excluded from further analyses. The study thus comprised 134 participants, who were from 14 of the 34 SSA countries. More than three quarters (*n* = 105/134, 78.4%) were from South Africa, Mozambique and Botswana, with the highest number of respondents (*n* = 75/134, 56%) from South Africa (Figure 1).

The study population comprised predominantly radiologists (*n* = 32/134, 23.9%) and general surgeons (*n* = 33/134, 24.6%). Of the 32 radiologists, 30 were diagnostic radiologists and two were interventionists. Most of the study participants (*n* = 93/134, 69.4%) practised at academic institutions. Sixty-five (48.5%) were consultant-grade staff, and 57 (42.5%) had five to ten years of work experience. Close to half (*n* = 65/134, 48.5%) of the respondents saw less than five HCC cases, and 91 (67.5%) reviewed less than five CT and/or MRI scans for HCC per month. Twenty-five (18.7%) participants reported that they did not see any HCC patients in their practice (Table 1).

**TABLE 1:** Participant characteristics (*N* = 134).

Variable	*n*	%
**Speciality**		
General radiologist	30	22.4
Interventional radiologist	2	1.5
Hepatobiliary surgeon	18	13.4
Transplant surgeon	1	0.7
General surgeon	33	24.6
Gastroenterologist	5	3.7
Physician	19	14.2
Oncologist	4	3.0
General practitioner	22	16.4
**Staff grade**		
Consultant	65	48.5
Clinical fellow	18	13.4
Registrar	32	23.9
Medical officer	19	14.2
**Years of practice**		
< 5	33	24.6
5–10	57	42.5
11–15	20	14.9
> 15	24	17.9
**Hospital setting**		
Academic	93	69.4
Private	14	10.4
Mixed academic or private	17	12.7
Other	10	7.5
**Number of HCC patients seen per month**		
0	25	18.7
< 5	65	48.5
5–10	37	27.6
11–20	4	3.0
> 20	3	2.2
**Number of CT and/or MRI for HCC seen per month**		
< 5	91	67.9
5–10	35	26.1
11–20	6	4.5
> 20	2	1.5

HCC, hepatocellular carcinoma.

In five of the seven knowledge-based questions, radiologists significantly outperformed non-radiologists. Most importantly, radiologists had higher levels of knowledge regarding the reasons for which LI-RADS^®^ was created (*p* = 0.007) and its ability to categorise malignant lesions on CE-imaging (*p* = 0.026). They were also better versed with the size criteria requirement (*p* = 0.003) and the concept of arterial phase enhancement (*p* = 0.004) required for diagnosing HCC (Table 2).

**TABLE 2:** Knowledge of LI-RADS^®^ among 134 medical doctors from sub-Saharan Africa.

LI-RADS^®^ concepts explored with respective correct responses	Correct response rate						*p*
	Radiologist (*n* = 32)		Non-radiologist (*n* = 102)		All respondents (*N* = 134)		
	*n*	%	*n*	%	*n*	%
LI-RADS^®^ was created to: Standardise the interpretation of imaging of patients at risk for HCC Facilitate data collection Develop a global system for reporting, for international use	21	65.6	39	38.2	60	44.8	0.007
LI-RADS^®^ targets the following at-risk patient population: Chronic HBV without cirrhosis Cirrhosis Prior history of HCC	21	65.6	68	66.7	89	66.4	0.913
LI-RADS^®^ cannot be applied to cirrhosis because of Budd–Chiari syndrome	18	56.2	37	36.6	55	41.4	0.045
LI-RADS^®^ cannot be applied to patients under the age of 18 years	15	46.8	33	32.4	48	35.8	0.135
LI-RADS^®^ LR-M category indicates a high probability of malignancy but is not HCC specific	21	67.7	44	43.6	65	49.2	0.026
Arterial phase hyperenhancement is defined as enhancement in arterial phase greater than liver, resulting in brightness greater than liver	28	90.3	61	59.8	89	66.9	0.004
A 10 mm–19 mm observation with arterial phase hyperenhancement and non-peripheral washout is a LI-RADS^®^ LR5 lesion	24	77.4	46	45.1	70	52.6	0.003

HCC, hepatocellular carcinoma; HBV, hepatitis B virus.

Clinicians and radiologists had similar levels of knowledge regarding the target patient population for LI-RADS^®^ (*p* = 0.913) and its limitation in the paediatric population (*p* = 0.135). However, it is noteworthy that 35.8% of the entire cohort were not aware that LI-RADS^®^ could not be used in patients younger than 18 years of age. Eighteen (56.2%) of the 32 radiologists who took part in this study did not know that LI-RADS^®^ could not be used in patients with liver cirrhosis resulting from Budd-Chiari syndrome.

Less than half (*n* = 46/134, 34.3%) of the study participants stated that their institutions used LI-RADS^®^ to report on HCC (Table 3). Thirty-nine (29.1%) participants mentioned that only 0% to 25% of their CT/MRI followed LI-RADS^®^ reporting standards. In this survey, the two main barriers to adopting LI-RADS^®^ were lack of consistency in reporting by radiologists (*n* = 60/134, 44.8%) and lack of familiarity (*n* = 37/134, 27.6%) with LI-RADS^®^ criteria.

**TABLE 3:** Current practices and LI-RADS^®^ utilisation reported by study participants.

Current practices and LI-RADS^®^ utilisation reported by study participants	Response rate					
	Radiologist (*n* = 32)		Non-radiologist (*n* = 102)		All respondents (*N* = 134)	
	*n*	%	*n*	%	*n*	%
What is the current system in your hospital for the reporting of liver lesions at high risk of HCC?						
LI-RADS^®^	23	71.9	23	22.6	46	34.3
No current system	7	21.9	67	65.7	74	55.2
Other	2	6.3	12	11.7	14	10.4
At your institution, what percentage of CT or MRI reports utilise LI-RADS^®^ for liver lesions at risk of HCC?						
0% – 25%	6	18.8	33	32.4	39	29.1
26% – 50%	4	12.5	6	5.9	10	7.5
51% – 75%	4	12.5	7	6.9	11	8.2
76% – 100%	7	21.9	6	5.9	13	9.7
Unsure/do not know	11	34.4	50	49.0	61	45.5
What are the main barriers to the widespread adoption of LI-RADS^®^ in your hospital?						
Radiologists not consistently reporting using LI-RADS^®^	12	37.5	48	47.1	60	44.8
Do not see enough patients with chronic liver disease	3	9.4	10	9.8	13	9.7
Clinicians are not interested to shift from current practise and use LI-RADS^®^	2	6.3	7	6.9	9	6.7
LI-RADS^®^ is too complex	3	9.4	3	2.9	6	4.5
Own unfamiliarity with LI-RADS^®^	10	31.3	27	26.5	37	27.6
Other	2	6.3	7	6.9	9	6.7

HCC, hepatocellular carcinoma; HBV, hepatitis B virus.

With regard to the attitudes of participating clinicians, most participants (78.3%) stated that LI-RADS^®^ would be their preferred reporting system for HCC (Table 4). However, 52.2% (*n* = 70/134) of the survey respondents either did not feel confident to use LI-RADS^®^ or did not use the system at all. As far as educational expectations were concerned, 55 (41%) agreed that routine use of LI-RADS^®^ in MDT meetings would further their understanding of HCC and enhance the utilisation thereof.

**TABLE 4:** Attitudes and perceptions regarding LI-RADS^®^ among 134 study participants.

Attitudes and perceptions regarding LI-RADS^®^	Response rate					
	Radiologist (*n* = 32)		Non-radiologist (*n* = 102)		All respondents (*N* = 134)	
	*n*	%	*n*	%	*n*	%
With reference to CT/MRI imaging of liver lesions at high risk of HCC: would you prefer radiology reports with or without LI-RADS^®^?						
With LI-RADS^®^	30	93.8	75	73.5	105	78.3
Without LI-RADS^®^	1	3.1	3	2.9	4	3.0
Do not know	1	3.1	24	23.5	25	18.7
How confident are you when using LI-RADS^®^ criteria?						
Very confident, actively use it and understand the criteria	3	9.4	14	13.8	17	12.7
Moderately confident: occasional questions during use	18	56.3	29	28.4	47	25.1
Not confident at all	8	25.0	20	19.6	28	20.9
Do not use LI-RADS^®^	3	9.4	39	38.2	42	31.3
Which of the following would be most suitable to improve your current understanding of LI-RADS^®^?						
Practise LI-RADS^®^ criteria during interdisciplinary meetings	6	18.8	49	48.0	55	41.0
Didactic teaching with workshop and presentation	12	37.5	25	24.5	37	27.6
Self-teaching by reading on Internet	1	3.1	7	6.9	8	6.0
Visual aids: brochures, posters, etc.	1	3.1	4	3.9	5	3.7
An application on a mobile device	11	34.3	16	15.7	27	20.1
Other	1	3.1	1	1.0	2	1.5

## Discussion

High-resolution CE abdominal imaging forms an integral part of diagnostic and treatment algorithms for HCC.^6,7,19^ Hence, LI-RADS^®^ has emerged as an essential tool to streamline and standardise communication in HCC MDT meetings.^8,9,10,11,12,14,19,21,22^ The global burden of HCC lies predominantly in SSA and South-East Asia, but paradoxically almost all the studies examining the knowledge and perspectives of doctors regarding LI-RADS^®^ come from HICs.^1,2,15,16,18,20^ Recently, the International Hepato-Pancreato-Biliary Association (IHPBA) identified HCC in SSA as a public health priority.^23^ Through a Legacy project, the IHPBA has drafted initiatives aimed at improving management pathways and access to care for HCC patients in SSA. One of the key components of this project includes the education of HCC MDT members.^24^ In line with the Legacy project, we conducted a multi-centre survey in SSA to examine the knowledge, current practices as well as the attitudes and perceptions of doctors regarding the LI-RADS^®^ system. The findings of this study will be used to develop educational initiatives for MDTs geared towards improving HCC treatment in SSA.

One hundred and thirty-four respondents from 14 SSA countries were included in the analysis, making it the most comprehensive study of its kind in the current literature. There were significant disparities in the knowledge of LI-RADS^®^ between radiologists and non-radiologists. Expectedly, radiologists performed better than clinicians in five of the seven knowledge-based questions. It is worthwhile noting that this survey explored basic LI-RADS^®^ concepts rather than specific imaging technicalities for HCC. As many HCC MDTs in SSA comprise mostly of general medical practitioners, the latter are expected to be familiar with basic LI-RADS^®^ concepts to ensure optimal communication throughout the treatment journey of these patients.^3,4,25,26^

Significant deficiencies in LI-RADS^®^ knowledge in both sub-groups were noted. A high proportion of radiologists (43.8%) and non-radiologists (63.4%) were unaware that LI-RADS^®^ cannot be applied to cirrhosis resulting from Budd–Chiari syndrome. Furthermore, more than half of the respondents in both groups failed to recognise that paediatric patients cannot be assessed using LI-RADS^®^. LI-RADS^®^ is typically not applied to cases of cirrhosis from vascular conditions because benign hyperplastic nodules can be misinterpreted as HCC lesions. LI-RADS^®^ has not been standardised in the paediatric population because of the low incidence of HCC in this sub-group of patients.^9,10,11,14^

In a nationwide survey from Germany, Ringe et al. examined the awareness and application of LI-RADS^®^ by consultants and trainees in tumour boards. Although 73.2% of the 77 respondents were aware of LIRADS^®^, only 19.2% used the classification when assessing patients with liver malignancy.^20^ These data are also echoed in another multi-centre North American study by Marks et al., where 48.7% of participating radiologists acknowledged that they did not use a formal reporting system for HCC during residency.^16^ In the current study, just over a third of all respondents stated that LI-RADS^®^ was the reporting standard for HCC at their respective institutions. These findings highlight the need for formal teaching and improved exposure to the LI-RADS^®^ classification system during residency training.

Although most clinicians were in favour of the routine use of LI-RADS^®^ in HCC care, the results of this survey showed that there are significant disparities in practice. Only a small number of clinicians (*n* = 17/134,12.7%) used the system in their daily practice, and 29.1% of the respondents reported that less than 25% of radiological reports were LI-RADS^®^ compliant.

Moreover, more than a quarter of participants (*n* = 37/134, 27.6%) reported a lack of familiarity with LI-RADS^®^ criteria, despite it being the overall preferred reporting system. Over half of study participants (*n* = 70/134, 52.2%) also stated that they did not feel confident using LI-RADS^®^ or did not use it at all.

Formal training of MDTs is a critical step in enhancing the use of LI-RADS^®^ globally. The survey participants were in favour of such an approach and considered the routine use of LI-RADS^®^ criteria at MDT meetings an effective means to boost their understanding thereof. These data are further supported by a survey of non-academic radiologists conducted by Marks et al., who showed that 60.9% of their radiologists suggested webinars and workshops to improve their LI-RADS^®^ knowledge.^16^ Among the 134 respondents in the current survey, the majority (93.3%) would adopt LI-RADS^®^ as their reporting standard for HCC. Perceived complexity of the LI-RADS^®^ system has been reported as a significant challenge in its adoption, a concern expressed by 12% to 14.5% of participants in large studies.^16^ In this SSA cohort, only 4.5% were concerned about the complexity LI-RADS^®^.

Lastly, a significant challenge in the widespread adoption of CT/MRI LI-RADS^®^ remains the paucity of high-field-strength MR scanners in SSA.^27,28,29,30,31^ In LI-RADS^®^, 1.5 Tesla (T) to 3.0T magnets are recommended in the diagnosis of HCC. Low magnetic field strength MRI is associated with a low signal-to-noise ratio, poor spatial resolution and inferior enhancement after gadolinium administration, which makes the detection of small HCCs difficult.^16,17^ More than 15% of North American radiologists have previously reported using less than 1.5T MR scanners.^16^ Future initiatives will need to focus on expanding the use of CE ultrasound and training thereof in the region.

### Limitations

A few study limitations are acknowledged. This study was voluntary and thus included only a proportion of doctors treating HCC in SSA. There is heterogeneity in the case volume, expertise and hospital infrastructure where patients are treated for HCC throughout SSA.^3,4,25,26^ Over half of the respondents (56%) were based in South Africa, which can limit the generalisability of the study findings to the broader SSA context. South Africa’s larger and more elaborate healthcare system and research infrastructure may not reflect the resource constraints, training exposure and imaging service availability in many other SSA countries. Thus, the results of this study should be interpreted contextually. These factors are potential confounders that can influence the knowledge and perspectives of the study respondents.

## Conclusion

This survey of clinicians and radiologists in SSA highlights the current knowledge, attitudes and perceptions regarding LI-RADS^®^. Timeous diagnosis of HCC is key to improving the prognosis of HCC on the African subcontinent. LI-RADS^®^ strives to achieve a common language for the definitive imaging diagnosis of HCC in high-risk patients globally. Facilitation of effective inter-disciplinary discussions using LI-RADS^®^ will ensure the early referral and management of patients with lesions at risk for HCC. The findings of the study will potentially influence and direct stakeholders to implement future educational interventions relevant to the subcontinent.
